# Novel blood product transfusion regimen to prevent clotting and citrate accumulation during continuous renal replacement therapy with regional citrate anticoagulation in children

**DOI:** 10.3389/fped.2023.1086420

**Published:** 2023-06-15

**Authors:** Yuelin Sun, Dong Li, Ke Bai, Feng Xu, Chengjun Liu, Hongxing Dang

**Affiliations:** Intensive Care Unit, Children's Hospital of Chongqing Medical University, Ministry of Education Key Laboratory of Children Development and Disorders, China International Science and Technology Cooperation Base of Child Development and Critical Disorders, Chongqing Key Laboratory of Pediatrics, Chongqing, China

**Keywords:** regional citrate anticoagulation, continuous renal replacement therapy, blood product transfusion, citrate accumulation, children

## Abstract

**Objective:**

Introduce a novel protocol to prevent clotting and citrate accumulation (CA) from blood product transfusion (BPT) during continuous renal replacement therapy (CRRT) with regional citrate anticoagulation (RCA) in children.

**Methods:**

We prospectively compared fresh frozen plasma (FFP) and platelet transfusions between the two BPT protocols, direct transfusion protocol (DTP) and partial replacement of citrate transfusion protocol (PRCTP), in terms of the risks of clotting, citric accumulation (CA), and hypocalcemia. For DTP, blood products were directly transfused without any adjustment to the original RCA-CRRT regimen. For PRCTP, the blood products were infused into the CRRT circulation near the sodium citrate infusion point, and the dosage of 4% sodium citrate was reduced depending on the dosage of sodium citrate in the blood products. Basic information and clinical data were recorded for all children. Heart rate, blood pressure, ionized calcium (iCa) and various pressure parameters were recorded before, during and after BPT, as well as coagulation indicators, electrolytes, and blood cell counts before and after BPT.

**Results:**

Twenty-six children received 44 PRCTPs and 15 children received 20 DTPs. The two groups had similar *in vitro* ionized calcium (iCa) concentrations (PRCTP: 0.33 ± 0.06 mmol/L, DTP: 0.31 ± 0.04 mmol/L), total filter lifespan (PRCTP: 49.33 ± 18.58, DTP: 50.65 ± 13.57 h), and filter lifespan after BPT (PRCTP: 25.31 ± 13.87, DTP: 23.39 ± 11.34 h). There was no visible filter clotting during BPT in any of the two groups. The two groups had no significant differences in arterial pressure, venous pressure, and transmembrane pressure before, during, or after BPT. Neither treatment led to significant decreases in WBC, RBC, or hemoglobin. The platelet transfusion group and the FFP group each had no significant decrease in platelets, and no significant increases in PT, APTT, and D-dimer. The most clinically significant changes were in the DTP group, in which the ratio of total calcium to ionized calcium (T/iCa) increased from 2.06 ± 0.19 to 2.52 ± 0.35, the percentage of patients with T/iCa above 2.5 increased from 5.0% to 45%, and the level of *in vivo* iCa increased from 1.02 ± 0.11 to 1.06 ± 0.09 mmol/L (all *p* < 0.05). Changes in these three indicators were not significant in the PRCTP group.

**Conclusion:**

Neither protocol was associated with filter clotting during RCA-CRRT. However, PRCTP was superior to DTP because it did not increase the risk of CA and hypocalcemia.

## 1. Introduction

Continuous renal replacement therapy (CRRT) is widely used to treat children who are critically ill with renal dysfunction and other serious conditions (1–4). Previous researches reported that about 4%–5% of critically ill children need CRRT (5, 6), often for more than 24 h. In addition, about 33%–65% of patients receiving CRRT need blood products transfusion (BPT) (7–9). Most researchers believed that BPT increases the risk of clotting in CRRT (10–12). However, Ramesh et al. (13) concluded that BPT had no significant relationship with the lifespan of the CRRT filter. So far, there is no consensus on whether or how BPT should be used during CRRT. In addition, most previous studies regarding BPT during CRRT used systemic heparin anticoagulation (SHA) (10–12), and only rarely used regional citrate anticoagulation (RCA). However, in 2012, the Kidney Disease Improving Global Outcomes (KDIGO) guidelines recommended RCA as the preferred anticoagulation protocol for CRRT (14). Therefore, it is necessary to study the influence of BPT in RCA-CRRT.

During RCA-CRRT, the circulating anticoagulation status is mainly related to the concentration of *in vitro* ionized calcium (iCa). Michael et al. found that blood does not clot when the concentration of iCa was below 0.33 mmol/L (15), and iCa itself is negatively correlated with the concentration of citric acid. Because BPTs increase the dosage of citric acid, they may theoretically not increase the risk of clotting. However, the body must metabolize this increased level of citrate, and patients may therefore have an increased risk of citrate accumulation (CA) and CA-related complications, such as hypocalcemia.

In theory, a stable citrate concentration will maintain a stable anticoagulant state, and the citrate concentrations in blood products are relatively constant. Thus, during RCA-CRRT, if the blood product is infused near the site where the sodium citrate is being pumped, and if the sodium citrate dose is reduced according to the amount in the blood product, it is possible to maintain a constant citric acid concentration, just as before BPT, and thereby achieve a stable iCa concentration and anticoagulant status. The research described here tested this hypothesis.

## 2. Methods

### 2.1. Patients

This prospective controlled study evaluated clotting and the risk of CA and CA-related complications in children receiving RCA-CRRT between different transfusion protocols. There were 44 PRCTPs in 26 children and 20 DTPs in 15 children, and the BPTs were platelets or fresh frozen plasma. We first conducted 44 sessions of PRCTP, and then conducted 20 sessions of DTP. Due to previous observation and theory, the CA risk of DTP is greater than that of PRCTP. For the safety of children, we selected more children with lower risk of CA, which was often predicted by smaller value of T/iCa, into the DPT group. All 41 children (age ≤18 years-old) who were admitted to the intensive care unit of the Children's Hospital of Chongqing Medical University and received transfusions of fresh frozen plasma (FFP) or platelets during RCA-CRRT from March 2021 to July 2022 were included. This study was approved by the hospital ethical review committee (Number: 2020.302).

### 2.2. Materials

A 4% citrate sodium anticoagulant solution (National Medicine Permit No. H20058913; Sichuan Nightingale Biological, China), which had a citric acid concentration of 136 mmol/L, was used.

FFP was anticoagulated using blood preservation solution II, and the calculated citrate concentration was about 15 mmol/L. Platelets were anticoagulated using blood preservation solution I, and the calculated citrate concentration was also about 15 mmol/L.

### 2.3. Methods

**Figure 1:**
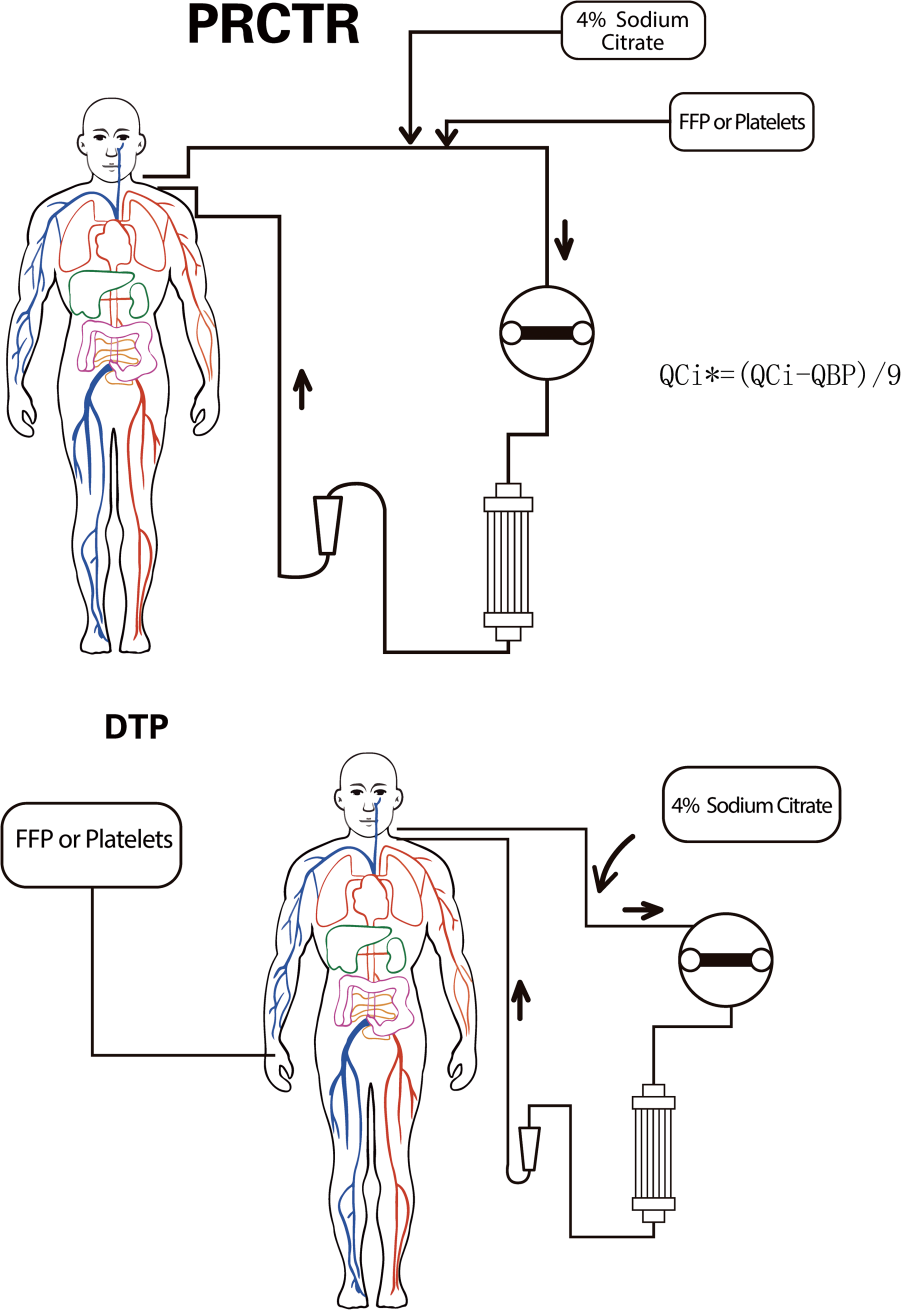
Overview of the DTP and the PRCTP. PRCTP, partial replacement of citrate transfusion protocol; QCi, the infusion rate of 4% Sodium Citrate before PRCTP; QCi*, the infusion rate of 4% Sodium Citrate during PRCTP; QBP, the infusion rate of blood product; DTP, direct transfusion protocol.

Patients received the direct transfusion protocol (DTP) or the partial replacement of citrate transfusion protocol (PRCTP) (Figure 1). The overall T/iCa before BPT was lower in the DTP group, but there were also individual cases with relatively high T/iCa. For DTP during RCA-CRRT, blood products were directly transfused without any adjustment to the RCA-CRRT regimen. For the PRCTP, blood products were transfused into the CRRT circulation near the citrate transfusion point; at the same time, the dosage of 4% sodium citrate was reduced based on the amount in the blood products (Figure 1).

Basic information (body weight, age, gender, and underlying diagnostic category), and clinical data (blood product type, volume, transfusion hours, duration of CRRT before and after BPT, and filter lifespan) were recorded for all patients. Heart rate; blood pressure; ionized calcium; various pressure parameters before, during, and after BPT; coagulation indexes [prothrombin time (PT), activated partial thromboplastin time (APTT), fibrinogen, and D-dimer]; electrolytes; and blood cell counts were recorded before and after BPT.

### 2.4. Statistical analysis

SPSS version 22.0 was used for statistical analysis. Count data were expressed as number and percentage, and the chi-square test was used for comparisons. Continuous data with a normal distribution were expressed as mean ± standard deviation, and a *t*-test was used for comparisons; continuous data with a non-normal distribution were expressed as median (interquartile range, IQR), and were compared using the non-parametric Mann–Whitney *U*-test. A *p* value below 0.05 was considered statistically significant.

## 3. Results

Twenty-six children received 44 PRCRTPs and 15 children received 20 DTPs (Table 1). At baseline, these two groups had no significant differences in age, weight, PRISM-III score, type of blood product (fresh frozen plasma vs. platelets), or gender.

**Table 1:** Characteristics of children who underwent RCA-CRRT with transfusion of a blood product using the PRCTP or the DTP^a^.

	PRCTP	DTP	*p*
Sessions	44	20	
Children	26	15	
Age, months	6.00 (1.00, 22.00)	40.00 (2.00, 122.00)	0.136
Males	21 (81%)	11 (73%)	0.788
Weight, kg	7.50 (4.00,12.00)	14.00 (5.00,36.00)	0.311
PRISM-III score	25.25 ± 3.91	26.15 ± 3.62	0.530
Type of blood product			
FFP	25	16	0.095
Platelets	19	4	0.095

PRCTP, partial replacement of citrate transfusion protocol, DTP, direct transfusion protocol, FFP, fresh frozen plasma.

^a^ Data are given as number, number (%), median (IQR), or mean ± SD.

The *in vitro* iCa during BPT was 0.33 (±0.06) mmol/L in the PRCTP group and 0.31 (±0.04) mmol/L in the DTP group, the total filter lifespan was 49.33 h (±18.58) in the PRCTP group and 50.65 h (±13.57) in the DTP group, and the filter lifespan after BPT was 25.31 h (±13.87) in the PRCTP group and 23.39 h (±11.34) in the DT group (Table 2). The two groups had no significant differences in these three parameters, and also had no significant differences in BPT rate, BPT duration, ratio of total calcium to ionized calcium (T/iCa) after BPT, and percentage of patients with T/iCa above 2.5 before or after BTP (all *p* > 0.05). There was no visible filter-clotting in any of the two groups during BPT. However, compared to the DTP group, the PRCTP group had a greater T/iCa before BPT (2.33 vs. 2.06 mmol/L, *p* = 0.001) and a smaller increase of T/iCa after treatment (0.00 vs. 0.45 mmol/L, *p* < 0.001).

**Table 2:** Effect of the PRCTP and DTP on filtration characteristics and calcium status^a^.

	PRCTP (*n* = 44)	DTP (*n* = 20)	*p*
BPT rate, ml/kg/h	11.48 ± 5.41	11.26 ± 3.62	0.884
Filter lifespan, h	49.33 ± 18.58	50.65 ± 13.57	0.785
BPT hours, h	1.45 ± 0.50	1.31 ± 0.53	0.322
Filter lifespan before BPT, h	22.66 ± 14.18	25.28 ± 8.75	0.476
Filter lifespan after BPT, h	25.31 ± 13.87	23.39 ± 11.34	0.615
Clotting during BPT, *n*	0	0	
External iCa during BPT, mmol/L	0.33 ± 0.06	0.31 ± 0.04	0.143
T/iCa before BPT	2.33 ± 0.29	2.06 ± 0.19	<0.001
T/iCa after BPT	2.33 ± 0.26	2.52 ± 0.35	0.038
Change of T/iCa after treatment	0.00 ± 0.23	0.45 ± 0.22	<0.001
T/iCa > 2.5 before BPT	11 (25.0%)	1 (5.0%)	0.085
T/iCa > 2.5 after BPT	9 (20.5%)	9 (45.0%)	0.07

PRCTP, partial replacement of citrate transfusion protocol, DTP, direct transfusion protocol, BPT, blood product transfusion, iCa, *in vitro* ionized calcium, T/iCa, ratio of total calcium to ionized calcium.

^a^ Data are given as mean ± SD or number (%).

We also analyzed the clinical data of patients in the PRCTP group (Table 3) and the DTP group (Table 4) before, during, and after the BPT. Analysis of each group indicated no significant differences in arterial pressure, venous pressure, transmembrane pressure, heart rate, mean arterial pressure, white blood cell count (WBC), red blood cell count (RBC), and hemoglobin (Hb) at these three periods.

**Table 3:** Clinical characteristics of patients before, during, and after PRCTP (44 transfusions in 26 children)^a^.

	Before	During	After	*p*
WBC ×10^9^/L	8.53 ± 5.65	–	7.98 ± 4.81	0.184
RBC ×10^12^/L	3.10 ± 0.60	–	3.10 ± 0.62	0.987
Hemoglobin, g/L	91.43 ± 18.00	–	90.70 ± 18.02	0.638
T/iCa	2.33 ± 0.29	–	2.33 ± 0.26	0.918
T/iCa > 2.5	11 (25.0%)	–	9 (20.5%)	0.800
iCa^2+^ < 0.9	0 (0%)	–	0 (0%)	1.000
iCa, mmol/L	0.36 ± 0.06	0.33 ± 0.06	0.37 ± 0.06	0.018
iCa *in vivo*, mmol/L	1.06 ± 0.07	–	1.07 ± 0.09	0.162
HCO^3−^, mmol/L	23.72 ± 5.74	18.48 ± 3.91	22.61 ± 3.83	<0.001
QB/BW, ml/kg/min	3.52 ± 0.70			
Arterial pressure, mmHg	110.59 ± 28.03	110.34 ± 27.13	111.29 ± 26.31	0.986
Venous pressure, mmHg	87.93 ± 27.05	88.39 ± 25.66	88.75 ± 24.83	0.989
Transmembrane pressure, mmHg	55.14 ± 17.31	58.27 ± 19.78	60.39 ± 17.25	0.474
Heart rate, bpm	146.70 ± 34.40	146.11 ± 34.53	146.18 ± 33.60	0.996
Mean arterial pressure, mmHg	61.20 ± 13.25	62.77 ± 15.41	65.25 ± 14.52	0.417

PRCTP, partial replacement of citrate transfusion protocol, WBC, white blood cell, RBC, red blood cell, T/iCa, ratio of total calcium to ionized calcium, iCa, *in vitro* ionized calcium, HCO^3−^, bicarbonate, QB, blood flow rate, BW, body weight.

^a^ Data are given as mean ± SD or *n* (%).

**Table 4:** Clinical characteristics of patients before, during, and after DTP (20 transfusions in 15 children)^a^.

	Before	During	After	*p*
WBC ×10^9^/L	7.76 ± 6.51	–	7.99 ± 5.79	0.797
RBC ×10^12^/L	3.21 ± 0.48	–	2.99 ± 0.51	0.059
Hemoglobin, g/L	92.35 ± 8.64	–	87.90 ± 13.94	0.135
T/iCa	2.06 ± 0.19	–	2.52 ± 0.35	<0.001
T/iCa > 2.5	1 (5.0%)	–	9 (45.0%)	0.008
iCa^2+^ < 0.9	0 (0)	–	3 (15%)	0.115
External iCa, mmol/L	0.34 ± 0.06	0.31 ± 0.04	0.33 ± 0.07	0.265
iCa *in vivo*, mmol/L	1.06 ± 0.09	–	1.02 ± 0.11	0.029
HCO^3−^, mmol/L	25.75 ± 4.96	19.64 ± 4.59	25.65 ± 4.93	<0.001
QB/BW, ml/kg/min	3.03 ± 0.64			
Arterial pressure, mmHg	112.85 ± 25.38	114.15 ± 27.36	115.80 ± 25.90	0.939
Venous pressure, mmHg	86.45 ± 24.87	88.50 ± 23.88	89.50 ± 23.56	0.920
Transmembrane pressure, mmHg	54.05 ± 15.69	58.55 ± 18.58	57.60 ± 16.83	0.682
Heart rate, bpm	133.15 ± 29.21	133.30 ± 28.67	133.90 ± 27.02	0.996
Mean arterial pressure, mmHg	59.90 ± 18.71	62.10 ± 18.74	63.50 ± 21.21	0.843

PRCTP, partial replacement of citrate transfusion protocol, WBC, white blood cell, RBC, red blood cell, T/iCa, ratio of total calcium to ionized calcium, iCa, *in vitro* ionized calcium, HCO^3−^, bicarbonate, QB, blood flow rate, BW, body weight.

^a^ Data are given as mean ± SD or *n* (%).

The DTP group had significant increases in T/iCa, percentage of patients with T/iCa above 2.5, a significant decrease of *in vivo* iCa, and a significant change in HCO_3_^−^ (all *p* < 0.05), however there were no significantly changes in these three indicators in the PRCTP group.

We then compared five coagulation indicators before and after transfusion with FFP or platelets in the PRCTP and DTP groups (Table 5). Patients in the PRCTP group who received FFP had significant declines in the PT and APTT, and a significant increase in fibrinogen (all *p* < 0.05); patients in the PRCTP group who received platelets had a significant decline in APTT and significant increases in fibrinogen and platelets (all *p* < 0.05). Patients in the DTP group who received FFP had significant declines in the PT and APTT, and a significant increase in fibrinogen (all *p* < 0.05); patients in the DTP group who received platelets had a significant increase of platelets (*p* < 0.05).

**Table 5:** Coagulation indexes and platelet levels before and after PRCTP or DTP with FFP or platelets^a^.

	Before BPT	After BPT	*p*
**FFP with PRCTP (25 transfusions)**			
PT, s	26.10 (20.95, 33.45)	18.30 (15.50, 22.65)	<0.001
APTT, s	96.62 ± 42.10	55.30 ± 29.91	<0.001
Fibrinogen, g/L	1.07 ± 0.65	1.35 ± 0.66	<0.001
D-dimer, mg/L	8.01 (3.04, 14.00)	5.61 (4.31, 12.90)	0.778
Platelets ×10^9^/L	63.88 ± 48.02	62.76 ± 53.59	0.808
**Platelets with PRCTP (19 transfusions)**			
PT, s	18.00 (15.70, 26.10)	17.80 (13.50, 20.40)	0.704
APTT, s	74.76 ± 50.49	71.06 ± 52.24	0.016
Fibrinogen, g/L	1.16 ± 0.66	1.27 ± 0.73	0.023
D-dimer, mg/L	6.15 (3.22, 9.02)	6.08 (3.45, 9.93)	0.965
Platelets ×10^9^/L	15.84 ± 8.12	109.47 ± 71.39	<0.001
**FFP with DTP (16 transfusions)**			
PT, s	24.20 (20.10, 31.60)	15.50 (13.20, 17.00)	<0.001
APTT, s	106.30 ± 63.90	56.97 ± 42.08	0.001
Fibrinogen, g/L	1.17 ± 0.72	1.82 ± 1.14	0.028
D-dimer, mg/L	6.70 ± 5.76	7.72 ± 6.54	0.142
Platelets ×10^9^/L	66.47 ± 55.45	59.33 ± 46.20	0.106
**Platelets with DTP (4 transfusions)**			
PT, s	19.40 (17.55, 20.95)	18.20 (16.30, 21.38)	0.564
APTT, s	49.70 (45.33, 147.68)	51.10 (39.30, 148.33)	0.564
Fibrinogen, g/L	0.62 (0.50, 1.37)	0.85 (0.56, 1.42)	0.885
D-dimer, mg/L	5.23 (3.95, 39.65)	3.82 (3.04, 39.77)	0.386
Platelets ×10^9^	7.00 (6.00, 15.50)	62.50 (48.25, 107.50)	0.020

BPT, blood product transfusion, FFP, fresh frozen plasma, PRCTP, partial replacement of citrate transfusion protocol, PT, prothrombin time, APTT, activated partial thromboplastin time, DTP, direct transfusion protocol.

^a^ Data are given as mean ± SD or median (IQR).

## 4. Discussion

CRRT is widely used as a rescue treatment for critically ill patients, but only a few studies examined BPT during CRRT, and most of these studies were anticoagulated with SHA (10–12). Many researchers previously believed that BPT increased the risk of clotting during CRRT (10–12). However, RCA is gradually replacing SHA as the preferred anticoagulation protocol during CRRT and there is little known about the influence of BPT on the risk of clotting and the best BPT protocol to be used during RCA-CRRT. In this study, we found that both DTP and PRCTP did not increase the risk of clotting during RCA-CRRT. However, DTP group had an increased risk of CA and hypocalcemia. But our novel PRCTP regimen significantly reduced the risk of CA and hypocalcemia.

There are differences in the anticoagulation principles and monitoring standards for SHA and RCA, so the influence of BPT on coagulation during CRRT should also theoretically be different between SHA and RCA. Because the transfusion of FFP and platelets increase the number of clotting factors or platelets, which is more likely to increase the risk of clotting than the transfusion of red blood cell, and the concentration of citrate contained in FFP or platelets is much higher than that in red blood cell suspension (about 1.5 mmol/L), this study we focused on FFP and platelet infusion. When using SHA, clinicians typically monitor anticoagulation status by measuring the activated coagulation time or the APTT, with targets of 1.5–2 times the normal values (16). During FFP transfusion, coagulation factors are added, and the coagulation function of the child will gradually improve. Thus, using the same heparin dosage will make it difficult to maintain the ideal anticoagulation target, and the clotting risk will inevitably increase. It is also difficult to increase the dose of heparin to maintain a stable anticoagulant state. Even with platelet transfusion, the increased number of platelets will activate the clotting system and the risk of clotting will also increase. However, when using RCA, clinicians often use the *in vitro* iCa concentration as the anticoagulation target (16). Michael et al. found that blood did not clot when the iCa concentration was below 0.33 mmol/L (15), and the *in vitro* iCa concentration is negatively correlated with the citric acid concentration. During RCA-CRRT, if BPT is performed without any adjustment, such as DTP regimen, although the number of coagulation factors or platelets will increase, citrate contained in blood products will increase the total dosage of citrate in the CRRT circulation compared to original RCA-CRRT and lead to further reduction of *in vitro* iCa. Theoretically, this may still maintain effective anticoagulation, or even better anticoagulation effect. The results of this study were consistent with our speculation. In our DTP group in this study, *in vitro* iCa was lower during DTP (0.33 ± 0.06 mmol/L) than those before (0.36 ± 0.06 mmol/L) and after DTP (0.37 ± 0.06 mmol/L).

Our results showed that neither PRCTP nor DTP increased the risk of clotting during RCA-CRRT. Firstly, both groups had *in vitro* iCa concentrations during BPT below 0.4 mmol/L, within the anticoagulation target range (15, 17, 18). Secondly, our filter lifespan was comparable to those reported in other children receiving RCA-CRRT (24.6–72 h) (19–23), and our two groups had no significant difference in total filter lifespan (PRCTP: 49.33 ± 18.58 h vs. DTP: 50.65 ± 16.62 h, *p* = 0.785). The filter lifespan after BPT was about 24 h (PRCTP: 25.31 ± 13.87 h, DTP: 23.39 ± 11.34 h). Thirdly, platelet transfusion with PRCTP or DTP did not increase APTT, PT, or D-dimer, and did not decrease fibrinogen. In addition, there were no significant decreases in platelets in the groups that received FFP transfusion with PRCTP or DTP. Fourthly, PRCTP and DTP led to no significant increases in arterial pressure, venous pressure, or transmembrane pressure during and after treatment. Therefore, PRCTP and DTP can both maintain effective anticoagulation during RCA-CRRT without increasing the risk of filter clotting, and our two groups had no significant difference in this outcome.

However, additional citric acid injection may increase the risk of CA, and our PRCTP is theoretically a good option to maintain effective anticoagulation without increasing the risk of CA. DTP increased the dosage of citrate contained in the blood products based on the original RCA-CRRT, so the risk of CA was higher than that of the original RCA-CRRT. To reduce the risk of CA, it is therefore necessary to reduce the dosage of citrate. If the citrate dosage is reduced by slowing down the citrate pump during DTP, this will lead to an insufficient citrate concentration in the CRRT circulation and iCa *in vitro*, thereby increasing the risk of clotting. However, when a blood product is transfused using our PRCTP, this maintains an effective *in vitro* iCa, which is similar to the level before PRCTP, and does not increase the risk of CA. The following two aspects may be responsible for this effect. First, we adjusted the flow rate of the citrate pump during PRCTP according to the calculated dosage of citrate in the blood product, so as to make sure the actual dosage of citrate infusing into the body during PRCTP was similar to the dosage before PRCTP. The citric acid concentration in 4% sodium citrate is 136 mmol/L, which is 9 times higher than those in the FFP (15 mmol/L) and platelets (15 mmol/L). Second, because the blood product infusion point is close to the sodium citrate infusion point, the actual dosage of citrate entering the body, that is the sum of the dosage of citrate in the blood product and the dosage of sodium citrate after adjusted, is similar to the total dosage of citric acid before PRCTP, the citric acid concentration in the filter was approximately equal to that before PRCTP.

Our PRCTP group had significantly lower risk of CA and hypocalcemia than our DTP group. During RCA-CRRT, CA is often manifested as increased T/iCa ratio, hypocalcemia and metabolic acidosis, and T/iCa above 2.5 is often used as the diagnostic criteria for CA (24, 25). After DTP, the T/iCa (2.06 ± 0.19 vs. 2.52 ± 0.35, *p* <0.001) and the incidence of sessions with T/iCa above 2.5 (5% vs. 45%, *p* = 0.008) increased significantly; the *in vivo* iCa (1.06 ± 0.09 mmol/L vs. 1.02 ± 0.11 mmol/L, *p* = 0.029) decreased significantly and the incidence of hypocalcemia also increased though without statistical significance. However, we found that PRCTP led to no significant increase in T/iCa, the incidence of patients with T/iCa above 2.5 and hypocalcemia. Furthermore, we chose more children with relatively lower T/iCa before BPT to be included in the DTP group (2.06 ± 0.19 mmol/L) than that in the PRCTP group (2.33 ± 0.29 mmol/L) to avoid serious complications related to CA, considering that DTP could theoretically lead to a greater risk of CA. The increase in T/iCa after BPT was significantly higher in the DTP group than that in the PRCTP group (0.45 ± 0.22 vs. 0.00 ± 0.23, *p* < 0.001). We speculate that if T/iCa in the DTP group was comparable to that in the PRCTP group, it would most likely lead to more significant increase in CA and hypocalcemia complications in the DTP group, or even severe hypocalcemia, which would greatly endanger these children. Therefore, we believe that PRCTP significantly reduced the risk of CA compared with DTP.

A limitation of our study is that T/iCa was significantly lower in the DTP group than in the PRCTP group before BPT, which meant that CA risk was lower in the DTP group than in the PRCTP group and made the data of the two groups less consistent. However, this was for the sake of the safety of children in the DTP group, and our study results also suggested that our concerns were reasonable. This study was also limited due to its short duration and the small number of cases.

## 5. Conclusion

Both PRTCP and DTP did not increase the risks of filter clotting during RCA-CRRT. However, the use of PRCTP significantly decreased the risk of CA and hypocalcemia when compared to DTP. PRCTP is a good innovative method for blood product infusion during RCA-CRRT, which does not increase the risk of clotting and CA-related risk.

## Data Availability

The original contributions presented in the study are included in the article, further inquiries can be directed to the corresponding author.
